# Impact of new technologies on stress, attrition and well-being in emergency call centers: the NextGeneration 9–1-1 study protocol

**DOI:** 10.1186/s12889-018-5510-x

**Published:** 2018-05-04

**Authors:** Janet Baseman, Debra Revere, Ian Painter, Scott Stangenes, Michelle Lilly, Randal Beaton, Rebecca Calhoun, Hendrika Meischke

**Affiliations:** 1 Department of Epidemiology, University of Washington, Northwest Center for Public Health Practice, 1107 NE 45th St., Suite 400, Seattle, WA 98105 USA; 2 Department of Health Services, University of Washington, Northwest Center for Public Health Practice, 1107 NE 45th St., Suite 400, Seattle, WA 98105 USA; 3 Department of Psychology, Northern Illinois University, Psychology-Computer Science Building, DeKalb, IL 60115 USA; 4 Department of Psychosocial and Community Health, University of Washington, Box 357263, Seattle, WA 98195-7263 USA; 5 Center for Child and Family Well-Being, University of Washington, Box 351525, Seattle, WA 98195-1525 USA

## Abstract

**Background:**

Our public health emergency response system relies on the “first of the first responders”—the emergency call center workforce that handles the emergency needs of a public in distress. Call centers across the United States have been preparing for the “Next Generation 9-1-1” initiative, which will allow citizens to place 9–1-1 calls using a variety of digital technologies. The impacts of this initiative on a workforce that is already highly stressed is unknown. There is concern that these technology changes will increase stress, reduce job performance, contribute to maladaptive coping strategies, lower employee retention, or change morale in the workplace. Understanding these impacts to inform approaches for mitigating the health and performance risks associated with new technologies is crucial for ensuring the 911 system fulfills its mission of providing optimal emergency response to the public.

**Methods:**

Our project is an observational, prospective cohort study framed by the first new technology that will be implemented: text-to-911 calling. Emergency center call takers will be recruited nationwide. Data will be collected by online surveys distributed at each center before text-to-911 implementation; within the first month of implementation; and 6 months after implementation. Primary outcome measures are stress as measured by the Calgary Symptoms of Stress Index, use of sick leave, job performance, and job satisfaction. Primary analyses will use mixed effects regression models and mixed effects logistic regression models to estimate the change in outcome variables associated with text-to-911 implementation. Multiple secondary analyses will examine effects of stress on absenteeism; associations between technology attitudes and stress; effects of implementation on attitudes towards technology; and mitigating effects of job demands, job satisfaction, attitudes towards workplace technology and workplace support on change in stress.

**Discussion:**

Our public health dependence on this workforce for our security and safety makes it imperative that the impact of technological changes such as text-to-911 are researched so appropriate intervention efforts to can be developed. Failing to protect our 9–1-1 call takers from predictable health risks would be similar to knowingly exposing field emergency responders to a toxic situation without following OSHA required training and practice standards assuring their protection.

## Background

Next Generation 9–1-1 (NG911) is a national initiative that requires all wireless carriers and providers of “interconnected” text messaging applications to support bi-directional transmissions between the public and emergency call centers, including short message service (SMS, also known as text messaging) from a cellular phone, email, Skype or instant messaging [1]. In addition, NG911 will expand emergency information sources to include receipt of, interaction with and transmission of streaming video, photo uploads, and automatic crash notifications by emergency call centers [2]. This change is congruent with changes in the landscape of information and communication technologies and the public’s expectations for sending a 9–1-1 text call or Twitter message during an emergency event [3]. NG911 capability will also improve access for deaf/hard of hearing populations and callers in a situation in which texting may be safer than calling, for example, during an active robbery.

The effectiveness of the police, fire fighters and EMS paramedics depends on the emergency call takers and dispatchers (telecommunicators or TCs) who are the “first of the first responders” [4]. TCs prioritize and triage incoming calls, collect information for first responders and issue medical instructions to callers while help is on the way. Each TC fields thousands of calls that are unpredictable in volume, length and content every year while handling five to six computer screens during a single 9–1-1 call. Shifts are lengthy, 10- to 24-h, non-standard (outside of 9:00 AM to 5:00 PM, Monday through Friday) and often coupled with mandatory overtime [5]. Our public safety relies on the TCs who must maintain the cognitive and emotional resources to perform complex, multiple tasks and make precise decisions with fateful consequences under life/death time pressures while staying calm [6].

### Stress and psychological and physical health

Like the first responders they support, TC work is characterized by high demand and routine exposure to workplace stressors that are associated with a range of psychological and physical health consequences [7]. TCs are primarily involved in the initial response involved in an emergency and rarely play a part in final resolution or outcome of the call which may be a source of stress itself. TCs not only feel responsible for the safety of the public but also perceive themselves as responsible for the safety of the dispatched personnel [8]. And despite lack of a direct physical exposure to trauma, TCs also experience duty-related distress that enhances risk for adverse psychological outcomes. Peritraumatic stress—defined as the responses during or immediately after trauma exposure—have been shown to predict depression and post-traumatic stress disorder (PTSD) among firefighters, police officers, emergency service personnel [9, 10]. Only recently has this same peritraumatic association with depression and PTSD been identified in the TC workforce [11, 12]. In a study of secondary trauma exposure, Troxell (2008) reported high levels of peritraumatic distress and a positive relationship between peritraumatic distress and secondary traumatic stress in 9–1-1 TCs [5]. Similarly, high levels of peritraumatic distress and a moderate, positive relationship between peritraumatic distress and PTSD symptom severity have been reported in TCs, suggesting that direct, physical exposure to trauma may not be necessary to increase risk for PTSD in this group of workers [12, 13]. However, few studies have focused specifically on the association between TC workplace stress and psychological health.

### Other stress outcomes and associations

Research on workplace stress in call centers has reported associations with attrition, burnout, absenteeism and early retirement. While limited to call centers in WA State, a recent survey found that 42% of TCs assess their job as “stressful to very stressful” and 60% reported their job as demanding (47%) or extremely demanding (14%), with no differences by gender or years of service, suggesting that stress levels do not abate over time [14].

The cost of this demanding workplace cannot be ignored. A 2012 survey found that 63% of emergency center managers associated job stress with TC turnover [15]. Although there is great variety in size, location and type, 83% of centers in a call center retention study reported an increase in the number of dispatched calls as compared to the previous three years while struggling with a national average annual turnover rate of 19% [16]. “Burnout” (a combination of exhaustion, cynicism, job detachment, a sense of ineffectiveness and lack of accomplishment or perceived lack of recognition/appreciation [17]) was identified as a primary cause of these lowered retention rates [16]. Research has also positively associated TC burnout with both sickness- and work-related absenteeism [18], an increased likelihood of lower job performance [19], depressive symptoms and lower job/life satisfaction [20].

Workplace factors, such as perceived manager support, sense of control over one’s schedule and social relationships with co-workers, including workplace climate characteristics such as bullying/harassment, can all influence stress levels and outcomes [18, 21, 22]. Individual characteristics, such as overcommitment (an exhaustive pattern of coping associated with the inability to withdraw from work demands) has been positively associated with high stress scores in TCs [14]. However, a variety of factors, both environmental and individual, may buffer the impact of negative outcomes associated with stress. Lifestyle factors, such as physical activity, and emotion-regulating skills can reduce the impact of environmental stressors as can work-life balance [21, 23]. In addition, individual characteristics and differences, such as coping strategies, self-efficacy, resiliency and compassion satisfaction can play a part in reducing or ameliorating high levels of work-related stress [24–29].

### Concerns about impact of NG911 technologies

To date, studies of technology adoption by emergency call centers have focused on the ability of new technologies to improve the speed of emergency response, patient health outcomes and mortality, and hospital costs [30]. “Technostress” is defined as stress created by adoption of information and communication technologies which can impact worker productivity, job satisfaction, commitment to the organization, and retention [31, 32]. Another issue specific to text-to-911 calls is TCs reliance on verbal communication to obtain and convey critical, clear, time-sensitive information, as well as take the caller’s language, voice, tone and auditory background and surroundings into account in order to successfully delivering lifesaving information [33]. Research has found that when communication challenges affect efficient information gathering, TC stress can be exacerbated [34, 35]. Other NG911 implementation impacts of concern are possible health impacts [33]. While 9–1-1 TCs will receive training to adapt to the new technologies deployed through the NG911 initiative, the focus is solely on technical skills, not human resource skills such as how to handle psychological stress due to hyper-tasking, exposure to multi-media images (to which TCs have not historically been exposed and which may enhance peritraumatic distress and thus, heighten risk for PTSD), or cumulative stress effects that could increase risk for stress and mood-related disorders. Exposure to photo uploads of graphic physical injuries or streaming videos of fatalities on the Interstate may increase occupational stress and distress. In addition, it is unknown how performance might be impacted, for example, when a TC needs to issue character-limited text message instructions for clearing a blocked airway or administering CPR.

### Project aim

The “NextGeneration 911: From Multi-tasking to Hyper-tasking” Project is designed to systematically describe the impact of the NG911 technologies implementation on TC stress and job performance, focusing on the first NG911 technology to be implemented: text-to-911 calling. The project’s Specific Aim is to:

Measure levels of stress, job satisfaction and job performance among 9–1-1 TCs before and after the NG911 technologies implementation.

Study objectives include:

- Investigate the impact of new technologies and information sources on 9–1-1 TC work
- Understand moderators, mediators and buffers to stress in the emergency call center workplace
- Investigate the influence of stress on outcomes such as attrition, work-related absenteeism, and turnover intention
- Describe the roles of job demands, job controls, and job resources on stress

We plan to measure levels of stress among 9–1-1 TCs before and after implementation of text-to-911; explore variables of potential influence on stress, such as social support; and evaluate the relationship between those variables on stress-related outcomes such as sick days and intent to leave employment. We hypothesize that implementation of text-to-911, one of the NG911 technologies, will have a significant effect on TC stress levels across multiple indicators.

### Theoretical framework

Two conceptual frameworks inform the project approach: the Demands-Control model (DCM), currently considered one of the most influential models linking job characteristics (e.g., low levels of control and work overload) to job stress and stress-mediated health outcomes [36, 37] and the Job-Demands-Resources (JDR) Model which stipulates that both demands and resources (such as supervisor support) can have multiplicative or cumulative effects on job stress and stress-related outcomes such as attrition and turnover intention, burnout/exhaustion and absenteeism (illness and work-related) [38, 39].

The DCM focuses on the psychosocial job characteristics of job demands and job control (sometimes referred to as decision latitude) within the context of the interactions between a worker and the environment, and the importance of identifying extent of worker decision authority (control over work situation) and skill discretion (possibility of using skills and competencies). High demands and low control predict “high-strain” which leads to increased levels of depression, fatigue and cardiovascular disease and mortality. Workers with high control are buffered from the negative effect of demands on outcomes which leads to lower levels of illness [36]. Social support also can act as a buffer in high demand situations [40–42]. In the context of NG911 technologies implementation, the DCM suggests that a technology change such as text-to-911 will create a “High Strain” work environment with TCs at higher risk for elevated stress and related health problems which would influence the likelihood of higher turnover intention and absenteeism and reduce job satisfaction and job performance. However, TCs already work in a high strain work setting and adding more stressors could exacerbate an already taxed and burnt out workforce.

In addition, the DCM cannot explain why the same levels of demand and control in two workers of similar background can lead to differential behavioral or health outcomes [43] or that there are different types of demand, some of which may be positive, such as the challenge of learning a new skill [44, 45]. The JDR Model expands on the DCM by including resources: aspects of the workplace that help with achievement of work goals, reduce demands or stimulate growth and development. In this model resources of all types can buffer the impact of demands on stress outcomes [38] and also incorporates personal characteristics that contribute to resiliency to stress, such as self-efficacy, organizational-based self-esteem, and optimism, among others [39].

## Methods/design

### Study design

The NG911 Project is an observational, prospective cohort study. Between January 2015–June 2017 we will recruit TCs employed at emergency call centers nationwide to participate in a study to measure the impact of NG911 technologies implementation on worker stress, job satisfaction and job performance. Recruitment will continue until sample size requirements are met (see “Sample Size and Power Calculations” below). To address project aims, we include three data collection time points: baseline (before text-to-911 implementation); early implementation (within the first month of implementation); and 6 months after implementation. The first NG911 technology being widely implemented is text-to-911 and will be the focus of our study. Given text-to-911 will be deployed at different times at different call centers, data collection will be staggered and tailored to the implementation timeline at each participating center. The study has received Institutional Review Board approval (#49412, University of Washington [UW] Human Subjects Division, Seattle, WA) for all research activities.

### Study setting

As stated, NG911 technologies implementation is a phased process in emergency call centers across the country. Most call centers are beginning this process by enabling the text-to-911 functionality. Emergency call centers that anticipate deploying any NG911 technology during the study timeframe will be eligible to participate. Call centers already using any NG911 technology will not be eligible. All call center types will be eligible: primary public safety access points (PSAPs) which focus on police calls; secondary PSAPs (medical and fire); and centers that function as both primary and secondary PSAPs. Some emergency call centers employ uniformed staff (i.e., EMTs), however the majority of centers employ civilian TCs. We aim to include representation of TCs across small and large emergency call centers. Recruitment will begin in WA State and will expand as needed throughout the region and nationwide to ensure representation and diversity in the sample.

### Population

9–1-1 TCs working at any emergency call center will be eligible to participate. Given hiring requirements, no individuals under age 18 will be included. We will limit study participation to TCs which have completed training and are operating as fully functioning, independent TCs. TCs still in training will not be eligible.

Recruitment will be conducted in coordination with managers at the participating call centers. TCs will be recruited by memos, flyers and other outreach methods at call centers. Information about the study and invitations to participate will also be posted through national emergency call center forums. All recruitment materials will include information about the study, its purpose and methods, and study investigator contact information so potential subjects can ask questions about the study prior to enrolling.

### Sample size

Our primary outcome is symptoms of stress as measured by the Calgary Symptoms of Stress Index (cSOSI) [46]. Our aim is to measure changes in stress that occur with the introduction of NG911 technologies in call centers. Of primary interest are the comparisons between pre-implementation stress levels and post-implementation stress levels, and between the immediate post-implementation stress levels and long-term post-implementation stress levels.

Calculations of sample size were performed using standard sample size calculation software for a paired t-test [47]. Based on estimates of within-participant standard deviation from a longitudinal study using the cSOSI [48] sample size calculations assumed an effect size of 0.25 and a type I error rate of 5%, which will require 160 participants with data. Assuming an 18% loss to follow-up will require a total of 200 participants. With this sample size we estimate that we will have 80% power to detect a change in cSOSI score of 11 points. Previous studies have used an effect size of 0.4 as clinically meaningful [49–55].

### Study variables and measurement instruments

All instruments will be built using the University of Washington’s Catalyst survey tool (www.uw.edu/lst/web_tools/webq) which has been approved by the UW Human Subjects Division for creating anonymous or confidential surveys. Participation in the study will be voluntary and confidential.

The following instruments will be used to collect data:

- Enrollment Demographic Information. Participants will enroll online using electronic consent as approved by Human Subjects Review. Call center characteristics such as call center staffing data, absenteeism, etc., will be provided by managers (see Table 1A). TC (see Table 1B) and manager (see Table 1C) demographic information will be requested at enrollment and updated at each data collection point.
- Online TC Survey. The survey will be administered through a secure web site and will include items regarding personal stress from the cSOSI [46] which measures the type of and frequency with which respondents experience physical, psychological, and behavioral responses during a designated time frame selected by the investigator. The cSOSI is a self-report survey consisting of 56 items with 8 subscales: cardiovascular, muscle tension, neurological/GI, depression, anger, sympathetic arousal, upper respiratory and cognitive disorganization. The survey asks the frequency of occurrence of each item in the previous week using a 5 point scale (0 = never to 4 = very frequently). Both predictive and concurrent validity of the tool have been established in studies that captured effects of stress management therapies and correlation with levels of exposure to sources of occupational and illness related stress [48]. Participants will also be asked to complete survey items regarding job performance self-assessments, job satisfaction, impact of technologies (anticipated and actual) on performance, workplace support, and workplace demands (such as mandatory overtime) as described in Table 2.
- Online Manager Survey. At each data collection point managers will be asked to update call center information regarding absenteeism, vacancies, etc. as well as indicate number of hours they have had to fill in for TCs due to absenteeism, training or low census, their current work-related stress level and their perception of overall stress level at the call center as listed in Table 1A and C.

**Table 1:** Demographics to be Collected by Online Surveys Administered at Pre-NG911 (Baseline), During-NG911(within 1 month after deployment) and Post-NG911 Technologies Implementation (6 months after deployment)

1A. Call Center Work Environment Demographics & Characteristics		
Type of Center (urban/rural) # TCs Basic Shift Structure^b^ Shift Assignment Process^b^ Overtime Structure (voluntary/mandatory, # hours between shifts)^b^ Break Structure^b^ Benefits^b^	Training Protocols^b^ Absenteeism Level^a^ Attrition (Voluntary, Terminated, Laid Off, Retired)^a^ Current Vacancies/Vacancy Level^a^ Citizen Complaints/Inquiries^a^	Mandatory Overtime Required^a^ General Call Center Stress Level^a^ Call Center Technology-Related Stress Level^a^ Ongoing Training (topic, length)^a^
1B. TC Demographics & Characteristics		
Gender Age Range Age (years)^c^ Educational Level Race Ethnicity	Position/Role Years Worked as TC Current Shift/Work Schedule Partner Status Children under 18 Years Sick Days Taken (last month)	Vacation Taken (last month) Overtime, Mandatory & Voluntary (last month) Stressful Calls (last month) Regular Exercise Pet Ownership
1C. Manager Demographics & Characteristics		
Gender Age Range Age (years)^c^ Educational Level	Race Ethnicity Years Worked in Center Years as Manager in Center	Fill-in for TCs, # Hours (last month) Current Personal Work-Related Stress Level

^a^as reported by manager for last month ^b^captured by documentation review as needed ^c^final survey only

**Table 2:** Summary of Data to be Collected by Online Surveys Administered to TCs

2A. Primary Outcome Variables	
Construct/Variable	Definition
Stress	Symptoms of stress [46]
Job Satisfaction	Attitudes and feelings about work which can range from positive and favorable (indicating job satisfaction) to negative and unfavorable attitudes (indicating job dissatisfaction) [31, 58]
Job Performance: Perceived Usefulness & Utility	Degree to which a person believes that using a particular system would enhance his/her job performance (perceived usefulness) and would be free of effort (perceived ease of use) [59–61]
Job Performance: Job-Fit/Job Complexity	Utilization of a new tool or system as strongly influenced by perception of expected consequences (complexity of use and fit between the job and what the technology offers) [62]
Job Performance: Professional Efficacy	The extent to which a person believes s/he can meet workplace demands can mitigate the effect of high job demands on stress and stress-related burnout [39]
2B. Moderators	
Support Satisfaction	Perceptions of manager and co-worker social support as direct sources of stress as well as potential job resources and which influence appraisal of occupational stressors [38, 58, 63–65]
Network Conflict	Perceptions of manager and co-worker conflict as direct sources of stress and which influence appraisal of occupational stressors [65]
Multi-tasking	Having to handle several, multiple, possibly overwhelming tasks in a challenging environment with the potential of engendering task saturation. The technologies involved can influence perception of multi-tasking due to technology overload, uncertainty, and/or complexity [31, 32, 63, 66, 67]
Technologies & Task Load	Stress experienced by end users in organizations as a result of their use of ICTs, constant ICT changes and the physical, social and cognitive responses demanded by their use. Technostress is a combination of 5 different aspects or dimensions: techno-overload (ICTs force users to work faster and longer), techno-invasion (invasive effect of constantly being connected), techno-complexity (complexity associated with ICTs leads users to feel inadequate, forcing them to spend time and effort learning new skills), techno-insecurity (users feel threatened about losing their job due to ICTs) and techno-uncertainty (continual ICT changes and upgrades unsettle users and creates uncertainty about skills) [31, 32]
Overcommitment	An individual’s exhaustive coping style which can adversely impact the individual’s health [14, 66, 68]
Attitude toward Behavior	Positive or negative feelings about performing the target behavior [60, 69]
Turnover Intention	An employee’s intention to leave or stay at their current place of employment or current job role/title which is important in relation to job satisfaction, a salient antecedent of turnover intent and a key mediating variable between the work environment and turnover intent [70].
Absenteeism	Absence, distinguished by absence frequency, absence duration, and different types (illness-related sick leave vs work-related illness) [38, 64]
Specific Technologies	Perceptions of anticipated and actual impact of specific NG911 information sources on performance [71]
Work-Life Balance & Job Satisfaction	The extent to which an individual is engaged in and satisfied across different life roles—work role and family role—which is associated with quality of life and well-being, with a positive balance implying a high level 2of satisfaction and a negative balance suggesting a low level of satisfaction [72]
2C. Individual Characteristics & Open-Ended Items	
Current Overall Work-Related & Non-Work-Related Stress Levels Stress Coping Methods Self-Care/Outside of Work Support	Attitude towards Overtime Current & Preferred Position/Role Current & Preferred Shift/Work Schedule

### Statistical analysis

All analyses will be conducted in R [56].

#### Primary analysis

The study design involves repeated measures on participants at three time points. We will use multilevel models to examine changes in outcome measures over time and associations with technology changes. Primary outcome measures of interest are stress (cSOSI score), use of sick leave in the previous month, job performance, and job satisfaction (measure). The primary analyses will use a mixed effects regression models (for continuous outcomes) and mixed effects logistic regression models (for binary outcomes) to estimate the change in outcome variables associated with technology implementation. The models will include fixed effects for time (three levels; baseline, post implementation and follow up) and site, with a random intercept for participant. Association between baseline outcome measures on survey non-response for the post-implementation survey will be examined to assess if survey non-response is associated with the outcome measures. In the situation that a statistically significant association is observed we will conduct a sensitivity analysis to determine the extent to which the observed association can account for association between the outcome variables and implementation of technology by using multiple implementation to impute missing outcomes according to different levels of differential drop-out. Hypotheses will be conducted at the 0.05 level of significance with adjustment for multiple comparisons between time periods.

#### Secondary analyses

Multiple secondary analyses will utilize the same primary analysis protocol described above to address baseline effects of stress on absenteeism; baseline associations between technology attitudes and stress; effects of technology implementation on attitudes towards technology; mitigating effects of job demands, job satisfaction, attitudes towards workplace technology and workplace support on change in stress; and effects of survey time lag prior to and subsequent to implementation on stress and job satisfaction.

### Project schedule

The timing of the data collection at baseline, during and post-intervention will be determined by the staggered implementation of text-to-911 at the emergency call centers participating in the study. At each data collection point, study participants will be asked to complete a 30-min online survey around worker stress and demographics as described in “Study Variables and Measurement Instruments” above.

### Dissemination plan

The study’s findings will be submitted for publication in peer-reviewed journals and disseminated at national conferences and through emergency call center quarterly journals and social media. We will share the actual data only on the basis of agreement that the data will be used solely for research, that no individuals will be identified in any manner, that data will be secured by electronic safeguards, and that once data analysis is complete, the data will be returned or destroyed. In short, we plan to share the data in accordance with NIH policy.

## Discussion

This study will investigate how stress due to implementation of text-to-911 impacts TCs’ ability to cope with or accommodate to new technologies and the impact of new technologies on known stress outcomes, such as job dissatisfaction, fatigue, anxiety, overwork, overload and diminished work performance. In the 2002 NIOSH publication, “The changing organization of work and the safety and health of working people: Knowledge gaps and directions,” stress at work was recognized as a leading safety and health problem that requires focused research efforts [57]. Stressful and hazardous conditions such as exposure to psychological stress have implications for TCs’ work life quality, safety and health. Our public health dependence on TCs for our security and safety makes it imperative that the impact of technological changes such as the NG911 initiative are researched so appropriate intervention efforts to protect TCs can be developed. Failing to protect our 9–1-1 TC workforce from predictable health risks would be similar to knowingly exposing field emergency responders to a toxic situation without following OSHA required training and practice standards assuring their protection.

### Study limitations

A primary issue for this project is scheduling. Our recruitment schedule is predicated on timely deployment of NG911 technologies. Emergency call centers across the country have been anticipating this implementation for several years and budget and equipment selection decisions have routinely delayed the timeline. We believe we can overcome this limitation as all data collection instruments are available online so wide expansion of recruitment efforts is easily facilitated. In addition, deployment when it occurs has been initially limited to text-to-911 technologies so our study findings may be limited to stress impacts of this one technology rather than the entire suite of technologies included in the NG911 initiative.

It is possible that emergency call centers that elect to participate could introduce a selection bias in the subject sample. However, given the widely expressed concerns about overall NG911 technologies implementation, we believe any generalizability issues will be overcome by our recruitment strategy, which includes sampling from a variety of call centers that differ in organizational structure, size, service area and population coverage. An additional selection bias is that those who self-select into the study may represent levels of resilience or other personal psychological characteristics that differ from the general 9–1-1 TC workforce. However, we believe our recruitment strategy across different call centers will mitigate this generalizability issue. A final limitation is that newly hired TCs, as compared to seasoned workers, may be more comfortable with new technologies than the current workforce.

## Abbreviations

cSOSI: Calgary Symptoms of Stress Index
DCM: Demands-Control Model
ICT: Information and communication technology
JDR Model: Job-Demands-Resources Model
NG911: Next Generation 9–1-1
PSAPs: Primary public safety access points
PTSD: Post-traumatic stress disorder
SMS: Short message service
TC: Telecommunicator
UW: University of Washington
